# Influence of chitosan nanoparticles and fennel essential oils (*Foeniculum vulgare*) on the shelf life of *Huso huso* fish fillets during the storage

**DOI:** 10.1002/fsn3.1161

**Published:** 2019-08-19

**Authors:** Mohadeseh Maghami, Abbas Ali Motalebi, Seyed Amir Ali Anvar

**Affiliations:** ^1^ Department of Food Hygiene, Science and Research Branch Islamic Azad University Tehran Iran

**Keywords:** chitosan, essential oils, fish, nanoparticles, shelf life

## Abstract

Fish and fishery products are important parts of the human diet, but the microbial, chemical, and physical deteriorations limit their shelf life. Using the modified atmospheric packaging system and edible coatings is one of the main procedures to improve the shelf life of fish. In this research, the effect of chitosan nanoparticles (CNPs) loaded with fennel essential oils along with modified atmosphere packaging (MAP) system on chemical, microbial, and sensorial properties of *Huso huso* fish fillets during storage at fridge were evaluated. The results showed that coating fish fillets with CNPs and fennel EO significantly reduced the peroxide value, total volatile nitrogen, and thiobarbituric acid value compared with the control samples. Microbial analyses showed a lower number of mesophilic, psychotropic, pseudomonas, and lactic acid bacteria in coated fillets compared with control and MAP packaging. Fish fillets coated with CNPs and EO showed high acceptability in all sensorial attribute through the storage. It can be concluded that using CNPs and fennel EO along with MAP packaging can enhance the shelf life for *H. huso* fillets up to 18 days in the fridge.

## 1 INTRODUCTION

Fishes and fishery products play a significant role in the human diet due to their unique nutritional properties (FAO, 2014). The fishery products contain a high amount of digestible proteins, essential amino acids, health‐promoting polyunsaturated fatty acids (long chain omega 3 fatty acids), minerals such as calcium and iodine, and vitamins (Venugopal, 2005). Recently and due to awareness of consumers regarding the nutritional and safety issues attributed to other sources of proteins such as chicken and the beef, the consumption of seafood has increased worldwide (Jasour, Ehsani, Mehryar, & Naghibi, 2015).

The quality of fishery products includes the nutritional, microbial, biochemical, and physiochemical properties (Pietrowski, Tahergorabi, & Jaczynski, 2012). Postdeath degradation of fish due to various biochemical reactions (such as changes in protein and lipid content, and the formation of biogenic amines and hypoxanthine) and microbial spoilage makes them more perishable compared with other muscle foods (Matak, Tahergorabi, & Jaczynski, 2015).

Different methods such as lowering the storage temperature (Bahuaud et al., 2008; Fukuma, Yamane, Itoh, Tsukamasa, & Ando, 2012; Quitral et al., 2009), using vacuum packaging (Duun & Rustad, 2008; Uçak, Özogul, & Durmuş, 2011), modified atmosphere packaging (MAP) (Sivertsvik, Rosnes, & Kleiberg, 2003; Stamatis & Arkoudelos, 2007), and the addition of antioxidants and antimicrobial products in order to retard the fish deterioration have been proposed (Andevari & Rezaei, 2011; Atarés & Chiralt, 2016; Cai et al., 2015; Çoban & Patir, 2013; Karoui & Hassoun, 2017; Masniyom, 2011).

Edible films and coatings providing physical protection to protect the food products from different mechanical, chemical, and microbiological deteriorations which can be beneficial for seafood. Excellent moisture and oxygen barrier properties are crucial for edible films and coatings in seafood packaging. Polysaccharides, proteins, and lipids are three main materials used for this purpose (Dehghani, Hosseini, & Regenstein, 2018). Arfat, Benjakul, Vongkamjan, Sumpavapol, and Yarnpakdee (2015) used gelatin films for refrigerated sea bass slices, Dursun and Erkan (2014) used different proteins as edible coating for smoked fish, and Shokri, Ehsani, and Jasour (2015) studied the effect of whey protein films on the shelf life of rainbow trout fillets. Sodium alginate films are used for improving the shelf life of refrigerated bream (*Megalobrama amblycephala*) (Song, Liu, Shen, You, & Luo, 2011) and chitosan–gelatin film for extending the shelf life of fish fillets (Wu et al., 2014). Edible coating and films can be used as carriers of antimicrobial agents, antifungal agents, and antioxidants to reduce the microbial growth or chemical reactions on the fish surface (Falguera, Quintero, Jiménez, Muñoz, & Ibarz, 2011). Chitosan is a biopolymer derived from deacetylated chitin which obtains from exoskeletons of crustaceans and mollusks (Jasour et al., 2015). Chitosan is a nontoxic biodegradable coating and has wide application in preserving the seafood products due to antimicrobial and antifungal properties (Ojagh, Rezaei, Razavi, & Hosseini, 2010). The antimicrobial properties of chitosan depend on different factors including the type of chitosan, degree of polymerization, molecular weight, and pH. Chitosan nanoparticles have been synthesized as a drug carriers, gene carrier, drug delivery, and the release of active antimicrobial agents and antibacterial agents (Qi, Xu, Jiang, Hu, & Zou, 2004). Chitosan has been widely applied as an edible coating or film to improve the shelf life of fish and fishery products (Fan et al., 2009; Günlü & Koyun, 2013; Jeon, Kamil, & Shahidi, 2002; Nowzari, Shábanpour, & Ojagh, 2013; Soares, Mendes, & Vicente, 2013). Chitosan nanoparticles can be prepared by adding a poly‐anion such as tripolyphosphate (TPP) into the chitosan solution under steady stirring and can be used in drug delivery or gene therapy applications (Janes, Fresneau, Marazuela, Fabra, & Alonso, 2001).

The incorporation of chitosan and essential oils into food coating as a novel technique helps to inhibit the microorganism growth and to slow down the oxidation at the surface of food which leads to enhance the shelf life and improve the sensorial properties of the food product (Lekjing, 2016). Gómez‐Estaca, De Lacey, López‐Caballero, Gómez‐Guillén, and Montero (2010) used chitosan–gelatin biodegradable films to improve the shelf life of chilled fishes during the storage. Ojagh et al. (2010) improved the shelf life of rainbow trout fillets by the application chitosan and cinnamon EO. Hosseini, Rezaei, Zandi, and Farahmandghavi (2016) observed that the gelatin/chitosan nanoparticle films incorporated with *Origanum vulgare* L. essential oil exhibited excellent antimicrobial properties.

Fennel (*Foeniculum vulgare* L.) is a herb belonging to the *Apiaceae* family, and it cultivates widely in India, Egypt, and elsewhere for its strong flavored leaves and seeds. Fennel essential oil can be used as a new source of pharmaceutical materials for its antimicrobial and antioxidant activities (Roby, Sarhan, Selim, & Khalel, 2013). It has been reported that fennel essential oil is a potential natural bactericide to control plant and fungal bacterial diseases (Lo Cantore, Iacobellis, De Marco, Capasso, & Senatore, 2004). Diao, Hu, Zhang, and Xu (2014) reported antibacterial activity of fennel EO against *Staphylococcus albus*, *Bacillus subtilis*, *Salmonella typhimurium*, *Shigella dysenteriae*, and *Escherichia coli.*

The *Huso huso* is a species of anadromous fish in the *Acipenseriformes* family which live primarily in the Caspian and black sea basins, and it is an important natural source for its caviar and meat that is commonly processed into frozen fillet (Hosseini et al., 2010). Except for the microbial deterioration during the storage of fillets, changes in fish lipid can affect the flavor and texture of the flesh. Therefore, improving the shelf life of fish fillets by using different coating techniques and biological preservatives is interesting. The main goals of this research were to prepare chitosan nanoparticles and investigate the effect of nanoparticles with the fennel essential oil on the peroxide value (PV), total volatile nitrogen (TVN‐B), thiobarbituric acid (TBA), and microbial and sensorial analysis of *H. huso* fillets during storage at fridge.

## 2 MATERIALS AND METHODS

### 2.1 preparation of the fennel essential oil

Fennel seeds were purchased from a local market in Tehran, Iran. An amount of 50 g of the seeds was milled and placed in Erlenmeyer flasks for 24 hr with 500 ml of ethanol (95% w/w) to completely extract. Afterward, it was placed in a rotary evaporator and extraction was carried out at room temperature for 72 hr to prepare an adequate amount of the essential oil (Roby et al., 2013).

### 2.2 Chitosan nanoparticles preparation

Chitosan was dissolved at 1% and 1.5% (w/v) with 1% (v/v) acetic acid, and then, the pH raised to 4.6–4.8 by adding NaOH 10 N. In order to prepare nanoparticles, the same amounts of aqueous tripolyphosphate solution (0.25% w/v) and chitosan solutions were mixed on magnetic stirrer at 500 rpm and 50°C for 10 min. Afterward, the fennel essential oil (1%, v/v) was introduced into the CNPs solutions and mixed for more 15 min.

#### 2.2.1 Characterization of CNPs

Morphological characteristics of the chitosan nanoparticles were assessed using a high‐resolution Transmission Electron Microscope (TEM) device (TEM; CM120, Philips).

The zeta potential and particle size distribution of nanoparticles (1% and 1.5% w/v) were determined using a Zetasizer instrument (Nanotrac Wave, Microtrac) and dynamic light scattering (Nanotrac Wave, Microtrac), respectively.

### 2.3 Fish trail

*Huso huso* fishes were purchased at a local store in Tehran, Iran, and transported to the laboratory under refrigeration condition. Fish samples were cut into 72 fillets in the portion of 50–100 g each and shelf life studies of different treatments started on the same day the fillets prepared.

The following treatment groups were prepared: control batch without coating (Control), uncoated samples in MAP (map cntr), CNP‐loaded samples in MAP (map chit), fennel EO‐loaded samples in MAP (anise map), CNP‐ and EO‐loaded samples in MAP (map anise and chit). Fish fillets were kept in fridge 0–4°C for 27 days and the chemical, microbial, and sensorial properties of the treatments carried out on days 0, 3, 6, 9, 18, and 27.

For coatings, the fish fillets were immersed in CNPs and fennel EO solutions for 5 min and then dried in room temperature for 2 min to remove exceed solutions. MAP‐stored samples were kept in packaging system with 80% CO_2_, 19% N_2_, and 1% O_2._

### 2.4 Peroxide value determination

Peroxide value were determined according to the method described by Egan, Kirk, and Sawyer (1997) and the results expressed as m_eq_ of peroxide oxygen/kg fat. 50 g of fish samples were weighed into a 500‐ml beaker and blended with 60 ml of chloroform, 60 ml of methanol, and 30 ml of distilled water, and after 2 hr, it was separated in three phases. 20 ml of bottom phases (chloroform phase) was transferred to 250‐ml flask and mixed with acetic acid–chloroform (3:2). Afterward, 0.5 ml saturated KI and 30 ml water were added to a flask and allowed to stand with occasional shaking for 1 min. the solution was titrated with 0.01 N Na_2_S_2_O_3_ solution using 0.5 ml of 1% starch solution as an indicator until the blue color disappeared.

### 2.5 Total volatile base nitrogen determination

The total volatile base nitrogen (TVB‐N) content of fish was estimated according to the microdiffusion method of Goulas and Kontominas (2005) and expressed as mg TVB‐N per 100 g of fish flesh. In this method, MgO was added to homogenized fish samples. After the distillation of this mixture, it was collected in a flask containing boric acid solution (3% w/v). The mixed indicator was prepared from the dissolution of 0.1 g of methyl red and 0.1 g of methylene blue to 100 ml of ethanol. Afterward, the 0.05 M H_2_SO_4_ solution was used for titration of the boric acid solution and the consumption of sulfuric acid used as an indicator of TVB‐N value (mg N per 100 g of fish).

### 2.6 The thiobarbituric acid determination

The TBA content was determined according to the method of Kirk and Sawyer (1991) with some modifications. For this aim, 4 g of fillets was minced and added to 20 ml of trichloroacetic acid (TCA) (20% w/v) and mixed at 20,000 *g* for 2 min. 3 ml of the supernatant was introduced into 3 ml of 2‐trichloroacetic acid (0.1% w/v) and heated in a boiling water bath for 30 min, and after the cooling, the absorbance was measured at 532 nm by a spectrophotometer (Memmert, Model WNB14, Germany). The amount of TBA was expressed as mg of malondialdehyde per kg of fish flesh.

### 2.7 Microbial analysis

Ten grams of the fish fillets were mixed with 90 ml of sterile saline solution (0.09% w/v) and homogenized in a stomacher for 2 min. Then, the serial dilution was made for the following microorganism determinations: (a) Viable mesophilic bacteria were determined using plate count agar (Merck) after incubation for 24 hr at 37°C, (b) psychotropic bacteria were determined using Man, rogosa and sharpe (Macromedia PTY‐LTD) after 10 d of incubation at 4–7°C, (c) lactic acid bacteria were determined by MRS agar (Merck) for 48 hr at 37°C, and (d) *Pseudomonas were* determined using cetrimide agar (CA) for 2 days of incubation at 25°C.

### 2.8 Sensorial properties

A panel of 6 trained judges (four males and two female) from Islamic Azad University of Tehran were selected for sensorial analysis. Different sensory attributes such as odor, color, texture, and overall acceptance were evaluated using the 5‐point hedonic scale at different storage days (0, 3, 6, 9, 18, and 27).

### 2.9 Statistical analysis

The results were presented as mean ± standard deviation. One‐way analysis of variance (ANOVA) was carried out to test the effect of experimental conditions. SPSS version 16.0 (SPSS Statistical Software, Inc.) was used for statistical analysis. Duncan's post hoc test was used to assess the difference between pairs of means, and the difference at *p* < .05 was considered to be significant.

## 3 RESULTS AND DISCUSSION

### 3.1 Nanoparticles structure

**Figure 1:**
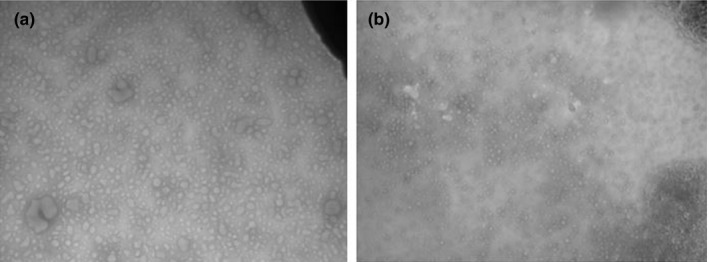
Transmission Electron Microscope pictures of chitosan nanoparticles with 1.5% (a) and 1% (b) of chitosan w/w

Transmission Electron Microscope was used to study the internal structure, morphology, and the size of CNPs. As it can be seen in Figure 1 by the increase in chitosan concentration, the size of CNPs increased, which that leads to aggregation and instability of nanoparticles. It can be concluded that the concentration 1% of chitosan is more appropriate for producing the nanoparticles to release the essential oils during the storage, and also, the smaller size of chitosan particles is probably more effective on the microbial cell wall (Ruparelia, Chatterjee, Duttagupta, & Mukherji, 2008). Gan, Wang, Cochrane, and McCarron (2005) reported typical shapes of polyhedrons (e.g., pentagon and hexagon) for CNPs that it was indicating a similar crystallization mechanism during the particle formation and growth process that suggests them as proper carriers for encapsulation and release mechanism.

The mean size and size distribution of nanoparticles were analyzed using the zetasizer analysis, and the size distribution profile is shown in Table 1. The results exhibited that the mean of NP diameter for 1% and 1.5% chitosan samples was 34 and 117 nm, respectively. Poly‐dispersity index (PDI) is another parameter to evaluate the stability of nanoparticles, and it decreased by the concentration of the chitosan such that the PDI for the 1% and 1.5% chitosan was 0.54 and 0.32, respectively. Oh, Chun, and Chandrasekaran (2019) observed the size of CNPs was in the diameter range of 100–1,000 nm and they also reported the potential antimicrobial activity of CNPs. Increase in CNPs size as a function of chitosan concentration was also observed by Gan et al. (2005). The method of preparing the nano‐chitosan can affect the PDI and stability of nanoparticles (Oh et al., 2019).

**Table 1:** The size distribution and poly‐dispersity index of chitosan nanoparticles

Sample	PDI	Particle size (nm)	Zeta potential
CNPs (1% w/w)	0.54	34	+41
CNPs (1.5% w/w)	0.32	117	+47

As it can be seen in Table 1, the zeta potential for 1% and 1.5% of chitosan was +41 and +47 mV, respectively. The positive charge for nanoparticles might be the result of the protonated amine groups in chitosan chains which results in high stability of chitosan nanoparticles and lower aggregations. Deen, Skovgaard, and Pedersen (2016) proposed that nanoparticles with the zeta potential least of +30 mV are more stable. The results showed the nanoparticles prepared from 1% chitosan were more stable compared with 1.5% chitosan and it can be due to the lower size of particles. Gan et al. (2005) also reported that the CNPs exhibited a positive charge and it increased by the increase in chitosan concentration.

### 3.2 PV determination

**Figure 2:**
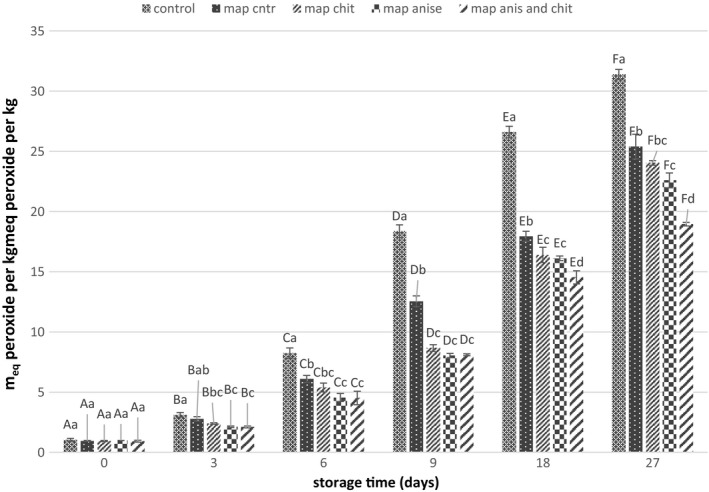
Changes in peroxide value of different samples during storage. Different letters of each bar indicate significant difference between the storage time within same analysis group (uppercase) and differences between treatment groups within same analysis day (lowercase) at *p* < .05

The peroxide test is a measure of the formation of hydroperoxides. PV measurement is used for indicating the primary oxidation products in the lipid fraction of the fish fillets (Eymard, Baron, & Jacobsen, 2009). PV for different treatments during storage is shown in Figure 2.

In this study, PV of fish fillets was in the range of 0.91 to 1.04 m_eq_/kg of fish lipid. It was observed that the PV in all treatments increased during the storage that it was the highest in the control sample so that, after 27 days of storage the number of primary oxidation products increased from 1.04 to 31.4 m_eq_/kg of lipid. No significant difference between PV for all treatments was observed during 6 days of storage while they were significantly lower compared with the control sample (*p* < .05). Although no difference between CNP‐coated treatments and fillets loaded with fennel EO was observed, the samples containing both EO and CNPs had the lowest PV, so that after 27 days of storage the PV was 18.95 m_eq_/kg of fish oil. The maximum permissible PV for fish oils is 5 m_eq_/kg (Piedrahíta Márquez, Fuenmayor, & Suarez Mahecha, 2019), from which all samples except the control and MAP were below the acceptable level, up to 6 days of storage.

It has been reported that EOs and other natural preservatives are able to reduce the oxidation reactions in meat products. Guran, Oksuztepe, Coban, and Incili (2015) observed that using EOs, such as thyme, sage, cloves, and rosemary in smoked rainbow trout, reduced the rate of hydroperoxide formation compared with control sample while there was no significant difference between the studied EOs in terms of reduction in PV. In fact, the phenolic compounds in the EOs by trapping the free radicals of the primary steps of oxidation (starting and propagation), through the formation of phenolic radicals, retard the spread of radicals and oxidation of fish oils (Maqsood & Benjakul, 2010). Sathivel, Liu, Huang, and Prinyawiwatkul (2007) reported that PV of frozen salmon fish coated with chitosan was lower compared with control samples.

### 3.3 TVN‐B determination

**Figure 3:**
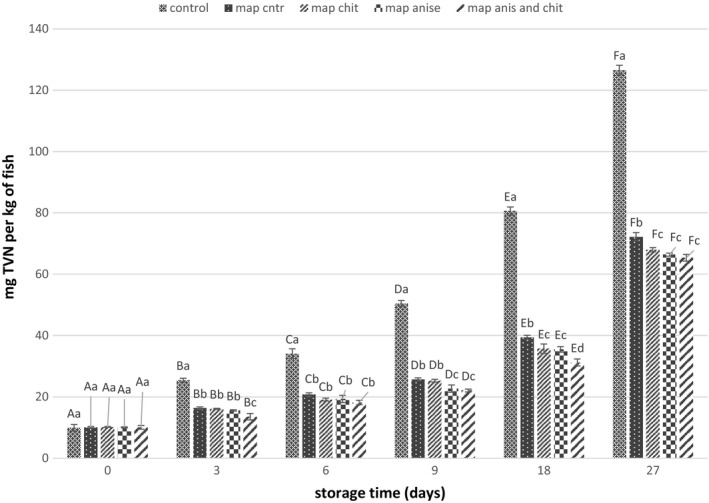
Changes in total volatile base nitrogen value of different samples during storage. Different letters of each bar indicate significant difference between the storage time within same analysis group (uppercase) and differences between treatment groups within same analysis day (lowercase) at *p* < .05

Total volatile nitrogen is widely used to determine the seafood quality since it has a direct relation with the microorganism growth and the formation of basic compounds resulted from their metabolism such as ammonia, trimethylamine, diethylamine and methylamine (Amin, 2012). TVN‐B values for the different treatments were in the range of 9.9 to 10.17 mg/kg of fish fillets on the first day, and it increased during the storage (Figure 3). the TVN‐B value for the control sample increased from 9.9 to 126.54 mg/kg fish, which it exceeded from the maximum level proposed for seafoods to consider as safe (35 mg/kg) after the 6th day of storage. It was observed that using the MAP technique was able to reduce the amount of nitrogen compound production during the storage compared with control and extend the shelf life up to 9 days (25.64 mg/kg). Coating the fillets with CNPs and fennel EO reduced the amount of TVN‐B during the storage time, but the difference between them was not significant (*p* > .05). It can be seen that the lowest TVN‐B value was observed in Map anise–CNPs treatment, so that at day 18 it was still at the acceptable level (14.53 mg/kg). It has been reported that MAP was able to control the TVN‐B value in sardine fish so that after 15 days of storage, it was 20 mg/kg (Özogul, Polat, & Özogul, 2004).

Piedrahíta Márquez et al. (2019) reported that the TVN‐B value in Cachama (*Piaractus brachypomus*) vacuum‐packed fish fillets that were coated with chitosan and propolis extract exceeded the acceptable limit (30 mg N/100 g) at day 16.

### 3.4 TBA determination

TBA is one of the indexes to illustrate the quality of fish, and it calculates from the malondialdehyde (MDA) content which the threshold level to percept the oxidation by the consumer is 0.5 mg malondialdehyde (4–10 MAD g/kg) per kg of fish (Sheard et al., 2000). In fact, the TBA represents the secondary oxidation products that affect the sensorial properties of fish (Hu, Wang, Xiao, & Bi, 2015).

**Figure 4:**
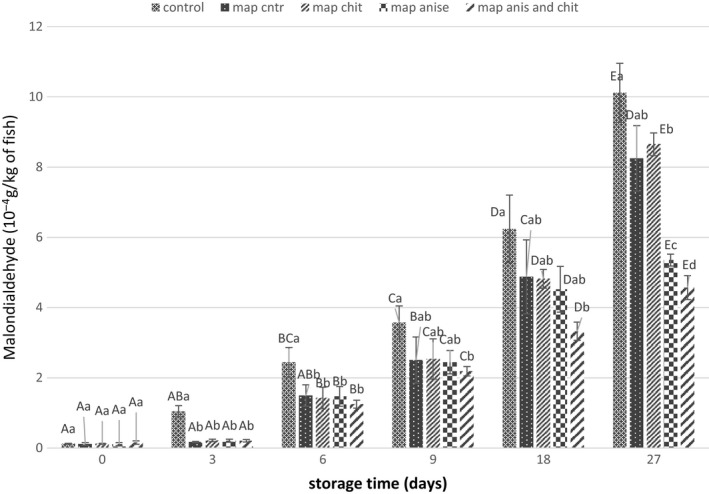
Changes in thiobarbituric acid value of different treatments during storage. Different letters of each bar indicate significant difference between the storage time within same analysis group (uppercase) and differences between treatment groups within same analysis day (lowercase) at *p* < .05

The TBA value in high‐quality food products should be less than 3 mg MDA/kg and in good quality material, not more than 5 mg MDA/kg (Kilinc, Cakli, Dincer, & Tolasa, 2009). Figure 4 shows the effect of different treatments on TBA formation of samples. As can be seen, the control sample had an acceptable level of MDA for 9 days of storage and it significantly increased after 18 days. In MAP sample, the amount of TBA was about 0.48 mg MDA/kg. The presence of fennel EO had a significant effect on TBA value. So that, after 18 days of storage the MDA content was 0.45 mg/kg of fish. After 27 days of storage, the amount of MDA in CNP‐coated samples was 0.88 while it was 0.53 mg/kg for fennel EO‐loaded samples. Application of CNPs coating with fennel EO significantly (*p* < .05) retarded the TBA production in the fish fillets and kept it in accepted level (0.45 mg MDA/kg). Different researches have also shown that EOs by having phenol compounds and tocopherols are able to retard the oxidation (Ariaii, Tavakolipour, Rezaei, Elhami Rad, & Bahram, 2015; Duan, Jiang, Cherian, & Zhao, 2010). It has been reported that the secondary lipid oxidation compounds in Cachama fish coated with chitosan–propolis was 12%–15% lower compared with control samples (Piedrahíta Márquez et al., 2019).

### 3.5 Microbial analysis

Table 2 shows the changes of mesophilic, psychrotrophic, *Pseudomonas, and* lactic acid bacteria (LAB) population during the storage period. The initial number of mesophilic bacteria on fish fillets was in the range of 2.18–2.35 log CFU/g, and it increased in all samples during the storage. The highest amount of mesophilic bacteria after 27 days of storage is observed in control samples (8.55 log CFU/g) while the Map anise and chit treatments showed the lowest number of bacteria (6.98 log CFU/g). It can be concluded that the different coating treatments were able to significantly delay the total mesophilic bacteria growth during the storage and synergism effect of CNPs and fennel EO was the most effective to reduce the mesophilic bacteria. Limited amount of accepted mesophilic bacteria on fish is 7 log CFU/g which it exceeded in control sample after 6 days, while the CNP‐ and EO‐loaded treatments were acceptable up to 18 days of storage. Petrou, Tsiraki, Giatrakou, and Savvaidis (2012) observed that application of chitosan coatings loaded with oregano EO were able to prevent the mesophilic bacteria growth to 3–4 log cycles compared with control. Pilehram, Roomiani, and Taddayoni (2018) reported that incorporation of the black mint extract in chitosan coating can reduce the microbial count in fish samples up to 2 log cycles. Storing the coated fish samples in MAP was also able to control the microbial number in acceptable level for 15 days.

**Table 2:** Microbial analysis of different treatments during storage period

Treatment	Days					
	0	3	6	9	18	27
Mesophilic						
Control	2.22 ± 0.08^Aa^	4.04 ± 0.17^Ba^	6.998 ± 0.20^Ba^	6.98 ± 0.20^Ca^	8.15 ± 0.11^Da^	8.555 ± 0.07^a^
Map cntr	2.19 ± 0.12^Aa^	3.21 ± 0.14^Bb^	4.21 ± 0.08^Cb^	5.72 ± 0.19^Db^	7.17 ± 0.15^Eb^	8.21 ± 0.10^Fb^
Map chit	2.21 ± 0.02^Aa^	3.24 ± 0.14^Bb^	4.03 ± 0.07^Cb^	5.25 ± 0.04^Dc^	6.76 ± 0.10^Ec^	8.25 ± 0.00^Fb^
Map anise	2.20 ± 0.12^Aa^	3.14 ± 0.04^Bb^	4.07 ± 0.01^Cb^	5.33 ± 0.09^Dc^	6.81 ± 0.13^Ec^	8.12 ± 0.16^Fb^
Map anise chit	2.20 ± 0.14^Aa^	3.06 ± 0.03^Bb^	4.03 ± 0.16^Cb^	5.23 ± 0.04^Dc^	6.17 ± 0.11^Ed^	6.98 ± 0.10^Fc^
Psychotropic						
Control	2.72 ± 0.16^Aa^	4.05 ± 0.19^Ba^	6.74 ± 0.23^Ca^	8.15 ± 0.05^Da^	8.38 ± 0.05^Ea^	8.55 ± 0.16^Fa^
Map cntr	2.71 ± 0.07^Aa^	3.31 ± 0.09^Bb^	5.86 ± 0.10^Cb^	5.72 ± 0.19^Db^	7.17 ± 0.0^Ea^	8.21 ± 0.06^Fa^
Map chit	2.72 ± 0.12^Aa^	3.31 ± 0.05^Bb^	4.94 ± 0.09^Cc^	5.25 ± 0.12^Dc^	6.76 ± 0.16^Eb^	8.25 ± 0.06^Eb^
Map anise	2.67 ± 0.07^Aa^	3.18 ± 0.05^Bb^	5.02 ± 0.09^Cc^	5.33 ± 0.03^Dc^	6.81 ± 0.14^Eb^	8.12 ± 0.22^Fb^
Map anise chit	2.72 ± 0.19^Aa^	3.14 ± 0.02^Bb^	4.30 ± 0.09^Cd^	5.23 ± 0.04^Dd^	6.17 ± 0.07^Ec^	6.97 ± 0.15^Fc^
Pseudomonas						
Control	2.04 ± 0.04^Aa^	3.46 ± 0.17^Ba^	4.65 ± 0.11^Ca^	5.99 ± 0.17^Da^	7.12 ± 0.05^Ea^	7.93 ± 0.19^Fa^
Map cntr	2.02 ± 0.16^Aa^	3.12 ± 0.10^Bb^	4.49 ± 0.15^Ca^	5.62 ± 0.1^Da^	6.88 ± 0.17^Ea^	7.70 ± 0.1^Fab^
Map chit	2.00 ± 0.02^Aa^	2.85 ± 0.09^Bb^	3.71 ± 0.09^Cb^	5.07 ± 0.06^Db^	6.18 ± 0.21^Eb^	7.19 ± 0.09^Fbc^
Map anise	2.02 ± 0.10^Aa^	2.94 ± 0.04^Bb^	3.84 ± 0.0^Cb^	5.08 ± 0.02^Db^	6.12 ± 0.11^Eb^	7.14 ± 0.16^Fc^
Map anise chit	2.02 ± 0.20^Aa^	3.34 ± 0.11^Bb^	4.07 ± 0.12^Cb^	5.20 ± 0.09^Db^	6.48 ± 0.10^Eb^	7.73 ± 0.20^Fc^
LAB						
Control	2.21 ± 0.06^Aa^	3.50 ± 0.08^Ba^	4.29 ± 0.03^Ca^	5.25 ± 0.14^Da^	6.56 ± 0.12^Ea^	6.75 ± 0.12^Fa^
Map cntr	2.18 ± 0.06^Aa^	3.24 ± 0.02^Bb^	4.27 ± 0.11^Ca^	5.14 ± 0.04^Dab^	6.17 ± 0.08^Eb^	6.17 ± 0.10^Fab^
Map chit	2.24 ± 0.09^Aa^	2.98 ± 0.05^Bc^	3.91 ± 0.07^Cb^	5.07 ± 0.05^Dab^	5.95 ± 0.12^Eb^	5.07 ± 0.15^Fab^
Map anise	2.24 ± 0.08^Aa^	3.06 ± 0.07^Bc^	4.19 ± 0.17^Ca^	4.89 ± 0.02^Dbc^	5.84 ± 0.11^Eb^	5.45 ± 0.09^Fab^
Map anise chit	2.21 ± 0.01^Aa^	2.93 ± 0.07^Bc^	3.71 ± 0.08^Cb^	4.74 ± 0.09^Dc^	5.93 ± 0.14^Eb^	4.65 ± 0.13^Fb^

Note

Different superscript lowercase letters (within each row) show differences between different treatments within same analysis day (*p* < .05). Different superscript uppercase letters (within each column) show differences between the storage time within same analysis group (*p* < .05).

Abbreviations: Control, control treatment; Map anise, fennel EO‐loaded samples that kept in MAP; Map anise chit, samples loaded with both CNPs and EO that kept in MAP; Map chit, CNP‐loaded samples that kept in MAP; MAP cntr, control samples kept at MAP.

Psychotropic bacteria such as *Pseudomonas*, *Alteromonas*, *Shewanella*, and *flavobacterium* are recognized as the dominant bacterium in fish fillets. As it can be seen in Table 2, the initial amount of psychotropic bacteria in different treatments was in the range of 2.68 to 2.72 log CFU/g and it increased during storage. So that, in the control samples it exceeded the threshold level (7.48 log CFU/g) after the 9th day of storage. Using MAP technique reduced the bacterial number compared with the control treatment, up to 1 log cycle. The difference between the CNP‐coated and EO‐loaded samples were not significant (*p* > .05) while their synergism effect was significant in reducing the psychotropic bacteria. Ojagh et al. (2010) also observed that using chitosan edible coating with EO was able to keep the psychotropic bacteria in accepted level up to 12 days of storage.

*Pseudomonas* are aerobic and gram‐negative bacteria that are considered as the most important causes of microbial deterioration in freshwater fishes (Cahill, 1990). These bacteria produce different compounds such as dimethyl sulfide, ketones, amines, and aldehydes and during the storage cause the putrefaction of fish (Dalgaard, 2003). As can be seen in Table 2, the growth of *Pseudomonas is* significantly influenced by the different treatments. Although the MAP technique was not effective enough to retard the *Pseudomonas* during the storage, the combined effect of MAP with CNPs and fennel EO significantly reduced the number of bacteria which it can be mainly attributed to the antibacterial effect of chitosan. The *Pseudomonas* population in control samples after 27 days of storage increased from 2 to 7.93 log CFU/g while in CNP‐loaded, fennel EO‐loaded, and the combination of them it was 7.09, 7.14, and 7.19 CFU/g, respectively. It can be concluded that the combination of CNPs and EO was not effective enough to retard the *Pseudomonas* during storage and it was effective only up to day 9. Using clove EO with gelatin–chitosan film drastically reduced the gram‐negative bacteria during storage (Gómez‐Estaca et al., 2010). Jeddi, Jafarpour, Yeganeh, and Naseri (2018) reported that there was no significant difference between chitosan‐coated and chitosan‐loaded Marjoram EO samples to retard the *Pseudomonas* growth. Hansen, Moen, Rødbotten, Berget, and Pettersen (2016) observed that using MAP can effectively retard the *Pseudomonas* growth on cod loins (*Gadus morhua*).

The results show that the LAB population increased in all treatments through 27 days of storage, which was the highest for control samples (Table 2). The initial population of LAB in different treatments was about 2.18–2.24 log CFU/g. No significant difference between control, MAP, and CNP‐coated samples during the storage was observed, while the fennel EO significantly reduced the LAB during the storage (*p* < .05). Similar results were observed for lamb treated with thyme EO and MAP, in which the treatment retarded the LAB growth during storage (Karabagias, Badeka, & Kontominas, 2011). It has been reported that LAB population on fish samples coated with gelatin–chitosan and clove EO remained stable through the storage (Gómez‐Estaca et al., 2010).

### 3.6 Sensorial analysis

**Figure 5:**
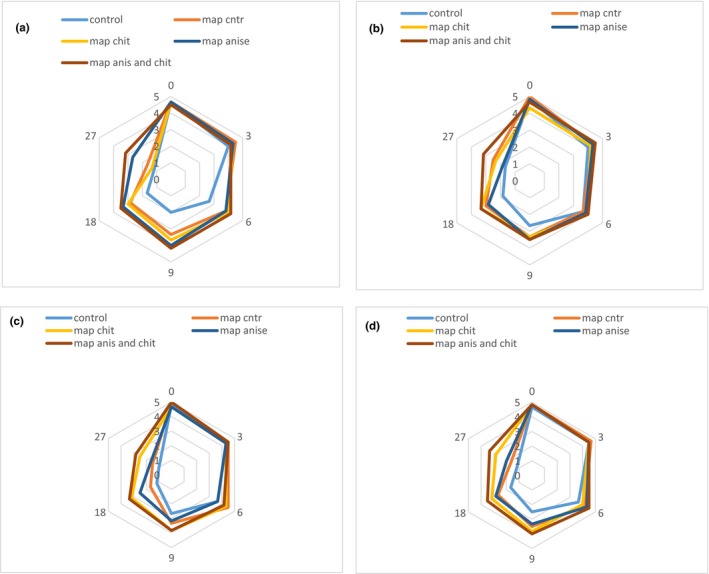
Sensorial properties of different treatments during storage: (a) odor, (b) color, (c) texture, and (d) overall acceptance

The results show that sensorial properties of samples were affected by different treatments and storage time (Figure 5). All samples had the acceptable sensorial attributes at the initial day of the storage but during the time and by the increase in spoilage, it declined. The flavor of fish significantly affects consumer acceptability. Fresh fishes are almost odorless which is related to the low quantity of volatiles (Venugopal, 2005). Control samples showed a low odor score after 6 days of storage, which it affirms observations for TVN‐B value. All treatments except the control were able to retard the spoilage odor in fish samples. Samples treated with CNPs and fennel EO showed the acceptable odor score after 18 days of storage and prohibited the amine and secondary oxidation products.

Color is another factor in fish quality, and it can be a sign of the freshness or spoilage of the fish (Mancini & Hunt, 2005). The color of the fish will change from pink to white which in some cases it can change to the darker color. Color changes in fish flesh is mainly due to oxidation and Millard reactions which presence an antioxidant can positively prohibit it (Ashie, Smith, Simpson, & Haard, 1996). The color score for all samples was acceptable for 9 days of storage. Samples coated with CNPs and EO had the highest score after days 18 and 27. Discoloration in fish samples can be due to oxidation and myoglobin changes (Chaijan, Benjakul, Visessanguan, & Faustman, 2005). Texture is an important quality parameter of muscle foods such as fish. Fish is generally softer than meat which is a result of low content of connective tissue and a lower degree of cross‐linking (Venugopal, 2005). As can be seen in Figure 5c, the presence of CNPs coating with or without EO was effective in retaining the texture of the fish up to 18 days of storage. Control sample received the lowest sensory score and after 9 days of storage, the texture score was 2.6.

Figure 5d shows that the overall acceptance of the samples declined through the storage. Control sample was acceptable to consumers up to 6 days and then after received low scores for overall acceptance. There was no significant difference (*p* > .05) between CNP‐coated samples with or without fennel EO during storage, and both were acceptable for 18 days of storage. Map anise and chit treatment had the highest overall acceptance at the day 27 (3.33) compared with other samples. Masniyom (2011) observed that the Asian sea bass (*Lates calcarifer*) fish samples loaded with oregano and thyme EOs were still fit for human consumption after 33 days of storage.

## 4 CONCLUSION

The aim of this research was to evaluate the effect of CNPs and fennel EO along with MAP technique on the shelf life of *Huso huso* fish fillets stored at the fridge. Hence, the chemical, microbial, and sensorial properties of fillets were studied during 27 days of storage. Chemical analyses such as PV, TBA, and TVN‐B showed that application of CNPs with fennel EO along with MAP could maintain the chemical factors of fish fillets in acceptable level while the control samples deteriorated after 6 days. Although the number of microorganisms increased in all treatments through the storage period, the number of pathogen bacteria was significantly lower in coated treatments compared with control. Treatment containing CNPs and EO had an acceptable level of microorganisms through the storage period. Sensorial properties of fish fillets also were affected by storage time and coating treatments. Study on odor, color, texture, and overall acceptance of samples showed that both of CNPs and fennel EO are able to improve the sensorial attributes of *Huso huso* fillets and increased the acceptance by the consumers. It can be concluded that using bioactive coatings with MAP improves the shelf life of *Huso huso* fish fillets up to 18 days.

## CONFLICT OF INTERESTS

The authors declared that they have no conflicts of interests.

## ETHICAL STATEMENTS

This study does not involve any human or animal testing.
